# Recurrence of Primary Aldosteronism After Surgery in Aldosterone-producing Adenoma With *KCNJ5* Gene Mutation

**DOI:** 10.1210/jcemcr/luac032

**Published:** 2023-01-11

**Authors:** Ko Aiga, Mitsuhiro Kometani, Daisuke Aono, Takashi Yoneda

**Affiliations:** Department of Health Promotion and Medicine of Future, Kanazawa University Graduate School of Medicine, Kanazawa, Ishikawa 920-8641, Japan; Department of Health Promotion and Medicine of Future, Kanazawa University Graduate School of Medicine, Kanazawa, Ishikawa 920-8641, Japan; Department of Health Promotion and Medicine of Future, Kanazawa University Graduate School of Medicine, Kanazawa, Ishikawa 920-8641, Japan; Department of Health Promotion and Medicine of Future, Kanazawa University Graduate School of Medicine, Kanazawa, Ishikawa 920-8641, Japan

**Keywords:** primary aldosteronism, aldosterone-producing adenoma, aldosterone-producing cell cluster, *KCNJ5*, adrenal venous sampling

## Abstract

Primary aldosteronism (PA) is a major cause of secondary hypertension. Aldosterone-producing adenoma (APA) is a subtype of PA, and adrenalectomy is the gold-standard treatment. Recently, a high prevalence of the *KCNJ5* gene mutation has been reported in APA, particularly in Japan. Herein, we present 2 extremely rare cases of PA recurrence more than 10 years after adrenalectomy for APA. In the first case, a 52-year-old woman was examined for hypertension 22 years after total adrenalectomy of the right adrenal gland. Recurrent PA was diagnosed based on high aldosterone-renin-ratio (ARR), identification of left adrenal gland tumor by computed tomography (CT), and a confirmatory test. In the second case, a 65-year-old man was examined for hypertension 17 years after total adrenalectomy of the left adrenal gland. He had maintained his blood pressure using medication since the onset of hypertension 4 years after the surgery. A year later, a high ARR was observed. PA recurrence was determined by a right adrenal gland tumor noted on CT and a confirmatory test. Somatic mutations in *KCNJ5* were detected in the resected tissues in both cases. We recommend careful follow-ups after adrenalectomy in APA cases, especially in those with a *KCNJ5* gene mutation.

Primary aldosteronism (PA) is a disease caused by autonomous excessive production of aldosterone. PA is characterized by a low level of serum potassium, suppression of plasma renin activity (PRA), and excessive production of aldosterone, which cause hypertension and severe cardiovascular diseases [1]. PA is a major cause of secondary hypertension and is classified into 2 subtypes: unilateral PA (aldosterone-producing adenoma [APA] or unilateral hyperplasia) and bilateral PA (bilateral adrenal hyperplasia) [1]. According to the Japan Endocrine Society, screening for PA is performed by evaluating the aldosterone-renin-ratio (ratio of plasma aldosterone concentration [PAC] and PRA) and PAC. Confirmatory tests are conducted to diagnose PA [1]. Although computed tomography (CT) is the most common modality to investigate the localization of PA, adrenal venous sampling (AVS) has higher sensitivity and specificity in determining the PA subtypes [1]. However, the Japan Endocrine Society recently suggested that patients younger than 35 years with common PA characteristics (ie, high PAC, unilateral adrenal tumor by imaging, and low serum potassium concentration) can directly undergo adrenalectomy without AVS [1]. The treatment of PA depends on the subtype. Medications, including mineralocorticoid receptor antagonists, are used for bilateral PA. There are several treatments for unilateral PA, of which adrenalectomy is the most preferred [1]. The recurrence of PA after successful surgical treatment for unilateral PA is extremely rare. To date, only 8 cases have been reported in the literature [2-6].

Given the improvement in exome-sequencing technologies, somatic driver mutations of APA have been identified and reported in the last decade. Today, somatic mutations in *KCNJ5*, *CACNA1D*, *ATP1A1*, *ATP2B3*, and *CTNNB1* are well known to drive the excessive production of aldosterone [7]. Among these genes, the prevalence of *KCNJ5* mutations in APA is high, particularly in Asian patients; 60% to 70% of patients with APA have mutations in *KCNJ5* [7]. Herein, we describe 2 cases of PA in patients with APA who underwent successful surgical treatment; however, PA recurred after 22 and 17 years in the first and second cases, respectively. In addition, *KCNJ5* mutations (G151R) were detected in the resected tissue in both cases.

## Case Presentation

### Case 1

A Japanese woman, who was age 29 years at the first presentation, had at that time presented with hypokalemia and hypertension. A medical examination revealed that her serum potassium level was 2.0 mEq/L, and the blood pressure was 214/140 mm Hg.

### Case 2

A Japanese man, who was age 47 years at the first presentation, had at that time presented with hypokalemia and hypertension. A medical examination revealed that his serum potassium level was 3.0 mEq/L, and the blood pressure was 240/140 mm Hg.

## Diagnostic Assessment

### Case 1

Laboratory data showed that the PRA was less than 0.15 ng/mL/h, and the PAC was 320 pg/mL. The diagnosis of PA was confirmed by the furosemide upright test and the rapid adrenocorticotropin (ACTH) stimulation test. No clinical and laboratory data suggested Cushing syndrome or other causes of secondary hypertension (Table 1). CT scan showed a right adrenal tumor 20 mm in diameter and a normal left adrenal gland. ^131^I-adosterol scintigraphy showed a significantly higher uptake in the right adrenal gland than in the left. In addition, AVS was performed, and the PAC in the right adrenal vein was high (19 130 pg/mL). Both adrenal veins were well selected (Table 2).

**Table 1.** Laboratory test data (case 1)

Test	29-year-old	52-year-old
Urine pH	6.0	6.0
AST, U/L	39	24
ALT, U/L	42	23
LDH, U/L	210	142
Na, mEq/L	144	142
K, mEq/L	2.8	4.3
Cl, mEq/L	104	106
Ca, mg/dL	—	9.0
IP, mg/dL	—	3.1
TP, mg/dL	—	6.9
BUN, mg/dL	12	16
Cr, mg/dL	0.8	0.6
Amylase, U/L	—	63
T-Cho, mg/dL	—	138
HDL, mg/dL	—	51
TGs, mg/dL	—	32
HbA_1c_, %	—	4.4
PAC, pg/mL	320	142
PRA, ng/mL/h	< 0.15	0.6
ARR	> 2133	237

Abbreviations: ALT, alanine aminotransferase; ARR, aldosterone-renin-ratio; AST, aspartate transaminase; BUN, blood urea nitrogen; Cr, creatinine; HbA_1c_, glycated hemoglobin A_1c_; HDL, high-density lipoprotein; IP, inorganic phosphorous; LDH, lactate dehydrogenase; PAC, plasma aldosterone concentration; PRA, plasma renin activity; T-Cho, total cholesterol; TGs, triglycerides; TP, total protein; UA, uric acid.

**Table 2.** Result of adrenal venous sampling

Case	IVC (PAC/F)	RAV (PAC/F)	LAV (PAC/F)
Case 1 (29-year-old)	233/303	19 130/2042	262/607
Case 2 (47-year-old)	170/248	131/248	5623/717

Abbreviations: F, plasma cortisol concentration (nmol/L); IVC, inferior vena cava; LAV, left adrenal vein; PAC, plasma aldosterone concentration (pg/mL); RAV, right adrenal vein.

### Case 2

Laboratory data showed that the PRA was 0.3 ng/mL/h, and the PAC was 137 pg/mL. The diagnosis of PA was confirmed by the furosemide upright test. No clinical and laboratory data suggested Cushing syndrome or other causes of secondary hypertension (Table 3). CT scan showed 2 left adrenal tumors with diameters of 14 and 13 mm, respectively. The uptake of ^131^I-adosterol in the left adrenal gland was not significantly higher than that in the right adrenal gland. In addition, AVS was performed, and the PAC in the left adrenal vein was high (5623 pg/mL), but the right adrenal vein was not well selected (see Table 2).

**Table 3.** Laboratory test data (case 2)

Test	47-year-old	65-year-old
Urine pH	6.0	6.0
AST, U/L	—	15
ALT, U/L	—	14
LDH, U/L	—	178
Na, mEq/L	145	145
K, mEq/L	3.0	3.6
Cl, mEq/L	104	108
Ca, mg/dL	4.4	9.2
IP, mg/dL	—	4.0
TP, mg/dL	—	7.0
BUN, mg/dL	—	15.6
Cr, mg/dL	1.1	0.79
Amylase, U/L	—	49
HDL, mg/dL	—	59
TGs, mg/dL	—	78
LDL, mg/dL	—	100
HbA_1c_, %	—	5.5
PAC, pg/mL	137	342
PRA, ng/mL/h	0.3	0.4
ARR	456	856

Abbreviations: ALT, alanine aminotransferase; ARR, aldosterone-renin-ratio; AST, aspartate transaminase; BUN, blood urea nitrogen; Cr, creatinine; HbA_1c_, glycated hemoglobin A_1c_; HDL, high-density lipoprotein; IP, inorganic phosphorous; LDH, lactate dehydrogenase; LDL, low-density lipoprotein; PAC, plasma aldosterone concentration; PRA, plasma renin activity; TP, total protein; TGs, triglycerides.

## Treatment

### Case 1

Based on the result of AVS, laparoscopic right adrenalectomy was performed.

### Case 2

Based on the result of AVS, laparoscopic left adrenalectomy was performed.

## Outcome and Follow-up

### Case 1

**Figure 1.**
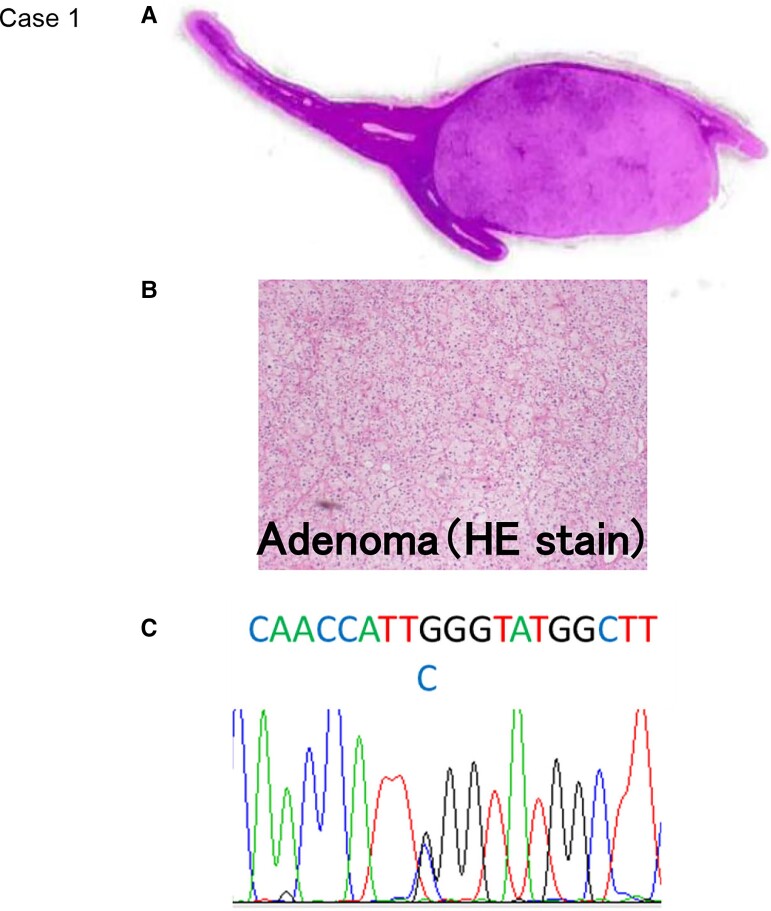
Histopathological and gene analysis (case 1). A and B, Hematoxylin-eosin (HE) stain of APA. C, Gene analysis of *KCNJ5* using the Sanger DNA sequencing technique. A mutation was detected in *KCNJ5*, and the change in the corresponding amino acid was confirmed (G151R).

**Figure 2.**
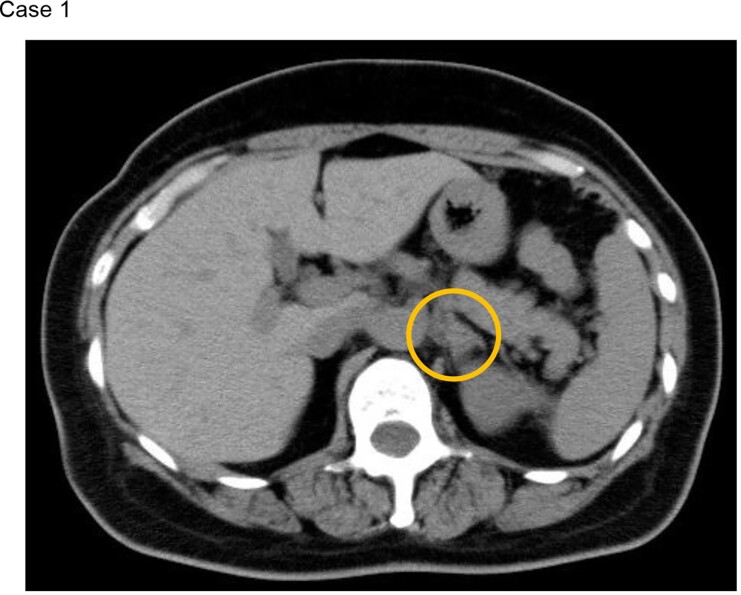
Image findings at the time of recurrence (case 1). The image shows computed tomography at age 52. The orange circle indicates a tumor with a diameter of 20 mm in the left adrenal gland.

After confirming the diagnosis, adrenalectomy was performed under 200 mg of spironolactone. After adrenalectomy, the patient's blood pressure normalized, and hypokalemia improved (Table 4). One month postoperatively, the PRA was 0.5 ng/mL/h, and the PAC was 95 pg/mL. The furosemide upright and rapid ACTH stimulation tests returned negative results. The pathological analysis of the adrenocortical tissue revealed the presence of an adenoma. We analyzed the APA for gene mutation. DNA was extracted from the adenoma and analyzed by Sanger DNA sequencing for mutations in the *KCNJ5* gene, and a G151R mutation was observed (Fig. 1). Twenty-two years after adrenalectomy, when the patient was age 52 years, she was referred to our hospital for hypertension again. Her blood pressure was 171/105 mm Hg; PRA, 0.6 ng/mL/h; and PAC, 142 pg/mL (see Table 1). The recurrence of PA was confirmed by the furosemide upright and captopril challenge tests. CT scan showed a left adrenal tumor with a diameter of 20 mm (Fig. 2).

**Table 4.** Data of hormones and blood pressure from preoperation to primary aldosteronism recurrence (case 1)

Case 1	Preoperation	Postoperation, after 1 mo	PA recurrence
BPs/BPd, mm Hg	214/140	107/70	171/105
Serum potassium, Eq/L	2.8	4.1	4.3
PAC, pg/mL	320	95	142
PRA, ng/mL/h	< 0.15	0.5	0.6
Antihypertensive drug	Free	Free	Free

Abbreviations: BPd, diastolic blood pressure; BPs, systolic blood pressure; PA, primary aldosteronism; PAC, plasma aldosterone concentration; PRA, plasma renin activity.

### Case 2

**Figure 3.**
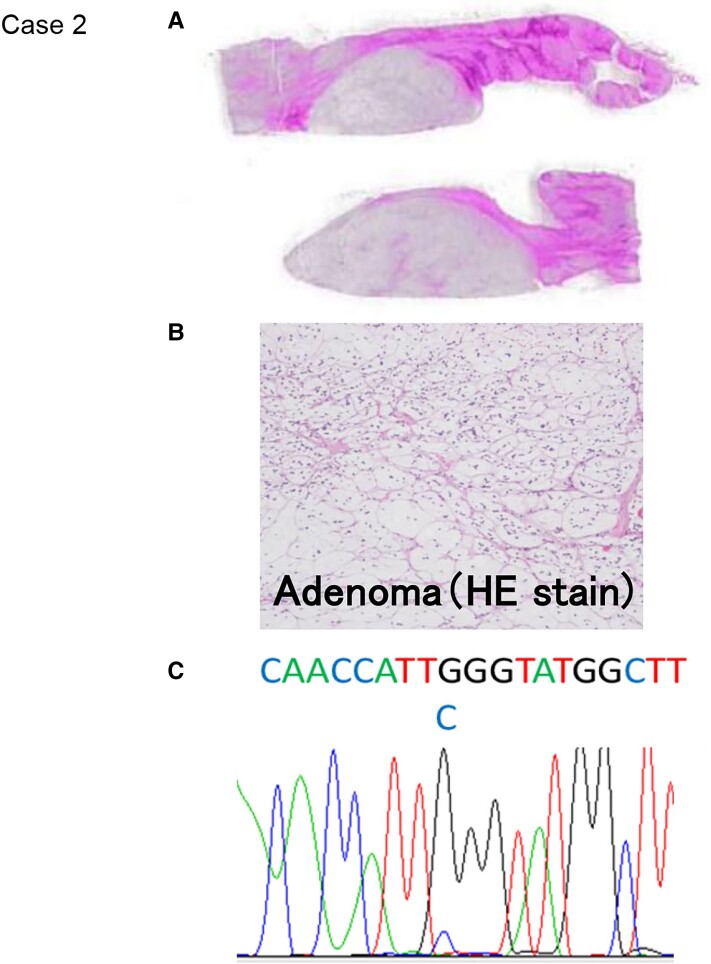
Histopathological and gene analysis (case 2). A and B, Hematoxylin-eosin (HE) stain of aldosterone-producing adenomas (APAs). C, Gene analysis of *KCNJ5* using the Sanger DNA sequencing technique. A mutation was detected in *KCNJ5* in both APAs, and the change in the corresponding amino acid was confirmed (G151R).

**Figure 4.**
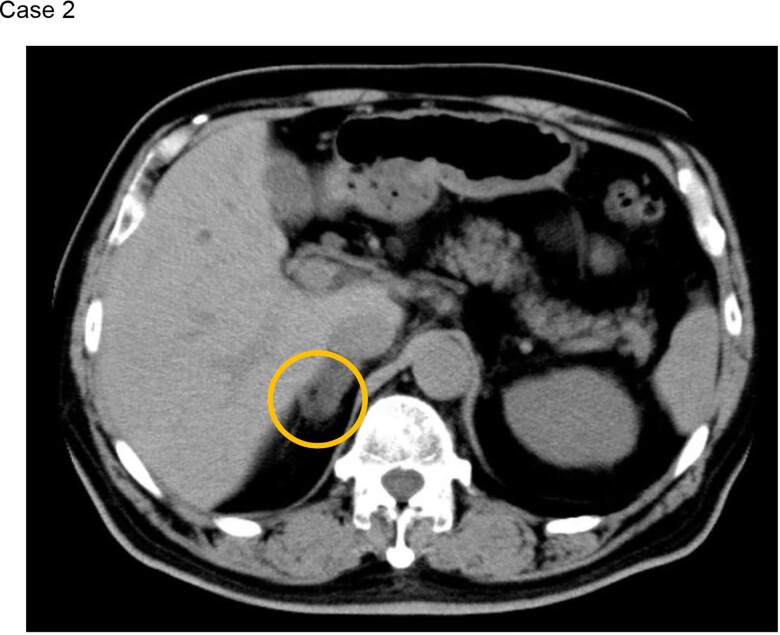
Imaging findings at the time of recurrence (case 2). The image shows computed tomography at age 65. The orange circle indicates a tumor with a diameter of 30 mm in the right adrenal gland.

Postoperatively, hypokalemia improved, and the patient's blood pressure normalized. His blood pressure was well controlled and maintained without medication after surgical treatment. The pathological analysis of the adrenocortical tissue revealed the presence of 2 adenomas. We analyzed these 2 adenomas for genetic mutations. DNA was extracted from the adenomas and analyzed for mutations in the *KCNJ5* gene by Sanger DNA sequencing, and the G151R mutation was observed in both adenomas (Fig. 3). Four years after surgical treatment, the patient's blood pressure increased again, after which it was controlled by medication (Table 5). After 5 years of surgical treatment, the ARR increased. The PRA was 0.6 ng/mL/h, and the PAC was 319 pg/mL. Seventeen years after the adrenalectomy, the recurrence of PA was confirmed by the captopril challenge test. CT scan showed a right adrenal tumor of 30 mm in diameter (Fig. 4).

**Table 5.** Data of hormones and blood pressure from preoperation to primary aldosteronism recurrence (case 2)

Case 2	Preoperation	Postoperation, after 5 y	Postoperation, after 10 y	Postoperation, after 15 y	PA recurrence
BPs/BPd, mm Hg	240/140	120/80	130/80	160/90	150/90
Serum potassium, Eq/L	3.0	3.5	3.5	3.8	3.6
PAC, pg/mL	137	319	292	137	342
PRA, ng/mL/h	0.3	0.6	0.1	0.3	0.4
Antihypertensive drug	Free	Enalapril (5 mg)	Amlodipine (5 mg)	Amlodipine (5 mg) Eplerenone (50 mg)	Amlodipine (10 mg) Doxazosin (2 mg)

Abbreviations: BPd, diastolic blood pressure; BPs, systolic blood pressure; PA, primary aldosteronism; PAC, plasma aldosterone concentration; PRA, plasma renin activity.

## Discussion

We described 2 cases of PA recurrence in the contralateral adrenal gland after 22 and 17 years of total adrenalectomy, respectively. Somatic mutations of G151R in the *KCNJ5* gene were detected in both cases by Sanger DNA sequencing.

Total adrenalectomy is considered the most effective treatment for patients with APA. However, there are other treatment options, such as perspicuous ethanol injection (PEI) and radiofrequency ablation (RFA) [1, 6, 8]. Treatments for APA are determined based on the situation. In general, successfully treated patients sustain their normalized blood pressure and serum potassium concentration after recovery from APA. PA recurrence is rare, and there are no treatment modalities established for patients with recurrent PA. To our knowledge, only 10 cases of PA recurrence, including our cases, have been reported, of which 7 had APA and 3 had unilateral or nodular adrenal hyperplasia [2-6]. Of these, gene analysis was conducted only in 3 cases. However, one of these cases did not undergo the confirmatory test or AVS to confirm the diagnosis of the first occurrence of PA [5]. Our 2 cases were the first ones to report somatic mutations in *KCNJ5,* accompanied by the results of screening, confirmatory tests, and lateralization techniques (CT and AVS). The recurrence site differs according to the treatment for patients with PA. Recurrence occurred in the contralateral adrenal gland after total adrenalectomy. In contrast, recurrence occurred in the ipsilateral adrenal gland after partial adrenalectomy or PEI [2-6]. Liu et al [8] investigated the long-term effects of APA in patients after RFA. However, PA recurrence was not observed (recurrent PA was zero out of 33 patients). In terms of recurrence, RFA may be a better treatment option for APA. However, the number of samples was insufficient, and follow-up durations were not long enough (∼ 6 years) to provide strong evidence.

PA recurrence in the ipsilateral adrenal gland after subtotal adrenalectomy or PEI could have resulted because the treatment was not fully effective on the entire range of PA sites. The untreated PA sites may have sufficiently developed over time to cause symptoms.

There are 2 reasons for PA recurrence at the contralateral adrenal gland after total adrenalectomy. The most plausible explanation is that it occurred independently, like the first PA. It can be considered that the niche of the adrenal gland is similar to that of the resected adrenal gland. To date, including our cases, mutations in *KCNJ5* have been detected in all the genetically analyzed recurrent PA cases, and all the cases were from Asia (Japan and China) [5]. The frequency of somatic mutations is known to be high, and germline mutation is rare in *KCNJ5*, indicating that this mutation is related to environmental factors [7]. Therefore, patients diagnosed with recurrent PA could have been susceptible to somatic mutations in the *KCNJ5* gene, and thus could explain PAs occurring in bilateral adrenal glands at different time points.

Another reason for recurrent PA is the aldosterone-producing cell cluster (APCC) to APA pathway. APCC is a small cluster present in the subcapsular adrenal cortex, which produces CYP11B2, but not CYP11B1 [9]. In contrast, the expression of CYP11B1 and CYP11B2 is commonly observed in APA [9]. Through the acquisition of the driver gene mutation, APCC possibly progresses to APA. Previous reports found mixtures of APA-like and APCC-like portions in some micronodules of patients with APA (APA-like portion: CYP11B1[+], CYP11B2[+]; APCC-like portion: CYP11B1[−], CYP11B2[+]). This supports the APCC-to-APA pathway [9]. In addition, the ACTH receptor (MC2R) was found to be overexpressed in APCC [9]. Kometani et al [10] reported a discrepancy in AVS in some patients with APA; PAC was significantly higher in the nondominant adrenal gland as determined by AVS without ACTH stimulation. This indicated the potential existence of APCC in the nondominant adrenal gland. The growth of a potential APCC into APA in the contralateral adrenal gland may have caused PA recurrence. Our 2 patients were diagnosed with APA through AVS without ACTH stimulation. However, an APCC may have existed in the contralateral adrenal gland, and the result of AVS with ACTH stimulation could have been different.

In conclusion, we present 2 cases of PA recurrence after total adrenalectomy. Somatic mutations in *KCNJ5* were detected in the extracted tissues of APA in both cases. PA recurrence is extremely rare, and there are no specific guidelines established for the management of recurrent PA. Early detection is crucial for the prevention of severe cardiovascular diseases. Long-term follow-up is recommended after the treatment of PA.

## Learning Points

- The driver genes of APA have been detected in previous studies. It is important to follow up patients with driver gene mutations detected in the resected APAs.
- The frequency and types of PA recurrence differ based on the treatments. It is necessary to investigate the association between PA recurrence and treatments.
- There are no specific guidelines for the management of patients with recurrent PA. Guidelines for managing the recurrence of PA are required.

## Data Availability

Restrictions apply to the availability of some or all data generated or analyzed during this study to preserve patient confidentiality or because they were used under license. The corresponding author will on request detail the restrictions and any conditions under which access to some data may be provided.

## Abbreviations

ACTH: adrenocorticotropin
ALT: alanine aminotransferase
APA: aldosterone-producing adenoma
APCC: aldosterone-producing cell cluster
ARR: aldosterone-renin-ratio
AST: aspartate transaminase
AVS: adrenal venous sampling
CT: computed tomography
PAC: plasma aldosterone concentration
PEI: perspicuous ethanol injection
PRA: plasma renin activity
RFA: radiofrequency ablation
